# 4-Methyl-*N*-phenyl­benzene­sulfonamide

**DOI:** 10.1107/S1600536809016377

**Published:** 2009-05-07

**Authors:** B. Thimme Gowda, Sabine Foro, P. G. Nirmala, Hiromitsu Terao, Hartmut Fuess

**Affiliations:** a Department of Chemistry, Mangalore University, Mangalagangotri 574 199, Mangalore, India; b Institute of Materials Science, Darmstadt University of Technology, Petersenstrasse 23, D-64287 Darmstadt, Germany; c Faculty of Integrated Arts and Sciences, Tokushima University, Minamijosanjima-cho, Tokushima 770-8502, Japan

## Abstract

In the title compound, C_13_H_13_NO_2_S, the dihedral angle between the aromatic rings is 68.4 (1)°. In the crystal, the molecules are linked into inversion dimers by pairs of N—H⋯O hydrogen bonds.  The unit cell of this compound was reported previously [Oh *et al.* (1985 ▶). *Chung. Kwa. Yong. (Chung. J. Sci.)*, **12**, 67] but no atomic coordinates were established in the earlier study.

## Related literature

For related structures, see: Gelbrich *et al.* (2007 ▶); Gowda *et al.* (2005 ▶, 2009 ▶ **a* ▶,b* ▶); Gowda, Foro, Nirmala, Terao & Fuess (2009 ▶); Perlovich *et al.* (2006 ▶).
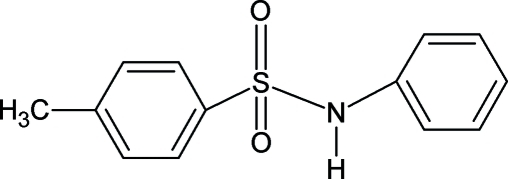


## Experimental

### 

#### Crystal data

C_13_H_13_NO_2_S
*M* *_r_* = 247.30
Monoclinic, 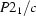

*a* = 8.770 (2) Å
*b* = 9.768 (2) Å
*c* = 16.234 (5) Å
β = 113.200 (2)°
*V* = 1278.2 (6) Å^3^
*Z* = 4
Cu *K*α radiation
μ = 2.17 mm^−1^
*T* = 299 K
0.55 × 0.50 × 0.40 mm

#### Data collection

Enraf–Nonius CAD-4 diffractometer
Absorption correction: ψ scan (North *et al.*, 1968 ▶) *T*_min_ = 0.336, *T*_max_ = 0.420
3091 measured reflections
2278 independent reflections
2041 reflections with *I* > 2σ(*I*)
*R*_int_ = 0.096
3 standard reflections frequency: 120 min intensity decay: 2.0%

#### Refinement

*R*[*F* ^2^ > 2σ(*F* ^2^)] = 0.071
*wR*(*F* ^2^) = 0.217
*S* = 1.10
2278 reflections
159 parameters
H atoms treated by a mixture of independent and constrained refinement
Δρ_max_ = 0.47 e Å^−3^
Δρ_min_ = −0.50 e Å^−3^

### 

Data collection: *CAD-4-PC* (Enraf–Nonius, 1996 ▶); cell refinement: *CAD-4-PC*; data reduction: *REDU4* (Stoe & Cie, 1987 ▶); program(s) used to solve structure: *SHELXS97* (Sheldrick, 2008 ▶); program(s) used to refine structure: *SHELXL97* (Sheldrick, 2008 ▶); molecular graphics: *PLATON* (Spek, 2009 ▶); software used to prepare material for publication: *SHELXL97*.

## Supplementary Material

Crystal structure: contains datablocks I, global. DOI: 10.1107/S1600536809016377/bq2138sup1.cif

Structure factors: contains datablocks I. DOI: 10.1107/S1600536809016377/bq2138Isup2.hkl

Additional supplementary materials:  crystallographic information; 3D view; checkCIF report

## Figures and Tables

**Table 1:** Hydrogen-bond geometry (Å, °)

*D*—H⋯*A*	*D*—H	H⋯*A*	*D*⋯*A*	*D*—H⋯*A*
N1—H1N⋯O1^i^	0.77 (4)	2.17 (5)	2.932 (4)	172 (4)

Symmetry code: (i) 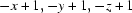.

**Fig. 1.**
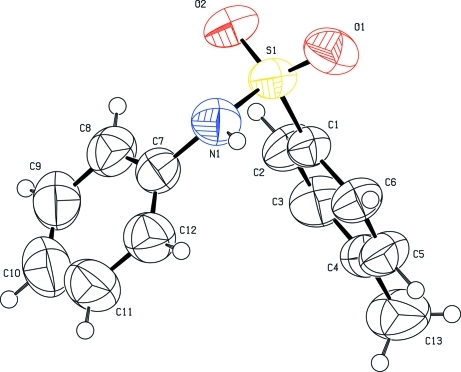
Molecular structure of (I), showing the atom labeling scheme and displacement ellipsoids drawn at the 50% probability level.

**Fig. 2.**
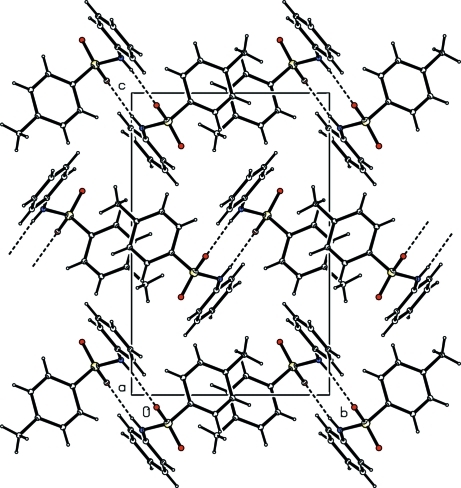
Molecular packing of (I) with hydrogen bonding shown as dashed lines.

## supplementary crystallographic information

### Comment

As part of a study of the effect of substituent on the crystal structures of *N*-(aryl)-arylsulfonamides (Gowda *et al.*, 2009*a, b, c*), in the present work, the structure of 4-methyl-*N*-(phenyl)- benzenesulfonamide (I) has been determined. The conformations of the N—C bond in the C—SO_2_—NH—C segment of the structure are "*trans*" and "*gauche*" with respect to the S══O bonds (Fig. 1). The molecule is bent at the S atom with the C—SO_2_—NH—C torsion angle of -51.6 (3)°. The two benzene rings in (I) are tilted relative to each other by 68.4 (1)°. The other bond parameters in (I) are similar to those observed in 2,4-dimethyl-*N*-(phenyl)-benzenesulfonamide (Gowda *et al.*, 2009 *a*), 4-chloro-2-methyl-*N*-(phenyl)benzenesulfonamide (Gowda *et al.*, 2009 *b*), 4-methyl-*N*-(3,4-dimethylphenyl)- benzenesulfonamide (Gowda *et al.*, 2009 *c*)) and other aryl sulfonamides (Perlovich *et al.*, 2006; Gelbrich *et al.*, 2007). The N—H···O hydrogen bonds (Table 1) pack the molecules into column like chains in the direction of *a*- axis (Fig. 2).

### Experimental

The purity of the commercial sample was checked and characterized by recording its infrared and NMR spectra (Gowda *et al.*, 2005). The single crystals used in X-ray diffraction studies were grown in ethanolic solution by slow evaporation at room temperature.

### Refinement

The N-bound H atom was located in difference map and its positional parameters were refined freely [N—H = 0.77 (4) Å]. The other H atoms were positioned with idealized geometry using a riding model [C—H = 0.93–0.96 Å] with *U*_iso_(H) = 1.2 *U*_eq_(*N*)

### Crystal data

**Table:**

C_13_H_13_NO_2_S	*F*(000) = 520
*M**_r_* = 247.30	*D*_x_ = 1.285 Mg m^−^^3^
Monoclinic, *P*2_1_/*c*	Cu *K*α radiation, λ = 1.54180 Å
Hall symbol: -P 2ybc	Cell parameters from 25 reflections
*a* = 8.770 (2) Å	θ = 5.4–20.7°
*b* = 9.768 (2) Å	µ = 2.17 mm^−^^1^
*c* = 16.234 (5) Å	*T* = 299 K
β = 113.200 (2)°	Prism, colourless
*V* = 1278.2 (6) Å^3^	0.55 × 0.50 × 0.40 mm
*Z* = 4	

### Data collection

**Table:**

Enraf–Nonius CAD-4 diffractometer	2041 reflections with *I* > 2σ(*I*)
Radiation source: fine-focus sealed tube	*R*_int_ = 0.096
graphite	θ_max_ = 66.9°, θ_min_ = 5.4°
ω/2θ scans	*h* = −3→10
Absorption correction: ψ scan (North *et al.*, 1968)	*k* = −11→0
*T*_min_ = 0.336, *T*_max_ = 0.420	*l* = −19→19
3091 measured reflections	3 standard reflections every 120 min
2278 independent reflections	intensity decay: 2.0%

### Refinement

**Table:**

Refinement on *F*^2^	Secondary atom site location: difference Fourier map
Least-squares matrix: full	Hydrogen site location: inferred from neighbouring sites
*R*[*F*^2^ > 2σ(*F*^2^)] = 0.071	H atoms treated by a mixture of independent and constrained refinement
*wR*(*F*^2^) = 0.217	*w* = 1/[σ^2^(*F*_o_^2^) + (0.1256*P*)^2^ + 0.4489*P*] where *P* = (*F*_o_^2^ + 2*F*_c_^2^)/3
*S* = 1.10	(Δ/σ)_max_ = 0.023
2278 reflections	Δρ_max_ = 0.47 e Å^−^^3^
159 parameters	Δρ_min_ = −0.50 e Å^−^^3^
0 restraints	Extinction correction: (*SHELXL97*; Sheldrick, 2008), Fc^*^=kFc[1+0.001xFc^2^λ^3^/sin(2θ)]^-1/4^
Primary atom site location: structure-invariant direct methods	Extinction coefficient: 0.060 (5)

### Special details

**Table:**

Geometry. All e.s.d.'s (except the e.s.d. in the dihedral angle between two l.s. planes) are estimated using the full covariance matrix. The cell e.s.d.'s are taken into account individually in the estimation of e.s.d.'s in distances, angles and torsion angles; correlations between e.s.d.'s in cell parameters are only used when they are defined by crystal symmetry. An approximate (isotropic) treatment of cell e.s.d.'s is used for estimating e.s.d.'s involving l.s. planes.
Refinement. Refinement of *F*^2^ against ALL reflections. The weighted *R*-factor *wR* and goodness of fit *S* are based on *F*^2^, conventional *R*-factors *R* are based on *F*, with *F* set to zero for negative *F*^2^. The threshold expression of *F*^2^ > σ(*F*^2^) is used only for calculating *R*-factors(gt) *etc*. and is not relevant to the choice of reflections for refinement. *R*-factors based on *F*^2^ are statistically about twice as large as those based on *F*, and *R*- factors based on ALL data will be even larger.

### Fractional atomic coordinates and isotropic or equivalent isotropic displacement parameters (Å^2^)

**Table:**

	*x*	*y*	*z*	*U*_iso_*/*U*_eq_	
C1	0.3623 (4)	0.2024 (3)	0.4713 (2)	0.0641 (7)	
C2	0.2930 (5)	0.0780 (4)	0.4369 (2)	0.0810 (9)	
H2	0.2768	0.0535	0.3787	0.097*	
C3	0.2480 (5)	−0.0098 (4)	0.4897 (3)	0.0929 (11)	
H3	0.2037	−0.0949	0.4670	0.112*	
C4	0.2669 (4)	0.0254 (4)	0.5752 (3)	0.0866 (11)	
C5	0.3360 (5)	0.1527 (5)	0.6073 (3)	0.0881 (11)	
H5	0.3495	0.1790	0.6648	0.106*	
C6	0.3838 (4)	0.2390 (4)	0.5571 (2)	0.0799 (9)	
H6	0.4313	0.3230	0.5804	0.096*	
C7	0.1172 (4)	0.4284 (3)	0.3472 (2)	0.0708 (8)	
C8	0.0374 (5)	0.3408 (4)	0.2771 (3)	0.0855 (10)	
H8	0.0972	0.2892	0.2519	0.103*	
C9	−0.1321 (5)	0.3311 (5)	0.2450 (3)	0.0982 (13)	
H9	−0.1867	0.2712	0.1981	0.118*	
C10	−0.2223 (5)	0.4065 (6)	0.2800 (4)	0.1095 (16)	
H10	−0.3372	0.3987	0.2570	0.131*	
C11	−0.1428 (6)	0.4932 (6)	0.3488 (4)	0.1133 (16)	
H11	−0.2042	0.5457	0.3725	0.136*	
C12	0.0292 (5)	0.5048 (4)	0.3844 (3)	0.0899 (11)	
H12	0.0833	0.5631	0.4324	0.108*	
C13	0.2197 (7)	−0.0689 (6)	0.6336 (4)	0.1236 (18)	
H13A	0.1824	−0.1544	0.6032	0.148*	
H13B	0.1322	−0.0283	0.6468	0.148*	
H13C	0.3142	−0.0845	0.6884	0.148*	
N1	0.2939 (3)	0.4457 (3)	0.3838 (2)	0.0762 (8)	
H1N	0.318 (5)	0.497 (5)	0.423 (3)	0.091*	
O1	0.5798 (3)	0.3739 (3)	0.45932 (19)	0.0863 (8)	
O2	0.4014 (3)	0.2496 (3)	0.32444 (16)	0.0858 (8)	
S1	0.42133 (9)	0.31592 (8)	0.40573 (5)	0.0689 (4)	

### Atomic displacement parameters (Å^2^)

**Table:**

	*U*^11^	*U*^22^	*U*^33^	*U*^12^	*U*^13^	*U*^23^
C1	0.0670 (15)	0.0712 (17)	0.0583 (16)	0.0062 (12)	0.0292 (13)	−0.0007 (13)
C2	0.104 (2)	0.077 (2)	0.071 (2)	−0.0127 (17)	0.0435 (18)	−0.0128 (16)
C3	0.111 (3)	0.077 (2)	0.103 (3)	−0.0077 (19)	0.055 (2)	0.001 (2)
C4	0.091 (2)	0.095 (2)	0.091 (2)	0.029 (2)	0.054 (2)	0.029 (2)
C5	0.103 (2)	0.106 (3)	0.066 (2)	0.017 (2)	0.0442 (18)	0.0030 (19)
C6	0.095 (2)	0.086 (2)	0.0664 (19)	0.0000 (17)	0.0399 (17)	−0.0132 (16)
C7	0.0718 (17)	0.0718 (18)	0.0711 (18)	−0.0004 (13)	0.0305 (14)	0.0198 (14)
C8	0.089 (2)	0.091 (2)	0.076 (2)	−0.0102 (18)	0.0330 (18)	0.0057 (18)
C9	0.084 (2)	0.106 (3)	0.090 (3)	−0.013 (2)	0.019 (2)	0.019 (2)
C10	0.079 (2)	0.121 (4)	0.114 (4)	−0.003 (2)	0.023 (2)	0.038 (3)
C11	0.094 (3)	0.127 (4)	0.128 (4)	0.030 (3)	0.054 (3)	0.028 (3)
C12	0.090 (2)	0.088 (2)	0.095 (3)	0.0093 (18)	0.0404 (19)	0.008 (2)
C13	0.143 (4)	0.120 (4)	0.140 (4)	0.033 (3)	0.090 (3)	0.052 (3)
N1	0.0754 (16)	0.0734 (17)	0.0824 (19)	−0.0075 (12)	0.0341 (14)	−0.0006 (13)
O1	0.0727 (13)	0.0901 (16)	0.1026 (18)	−0.0101 (11)	0.0414 (12)	−0.0152 (14)
O2	0.1054 (17)	0.0989 (18)	0.0709 (14)	−0.0013 (14)	0.0541 (13)	−0.0068 (12)
S1	0.0721 (6)	0.0749 (6)	0.0691 (6)	−0.0036 (3)	0.0379 (4)	−0.0048 (3)

### Geometric parameters (Å, °)

**Table:**

C1—C2	1.374 (5)	C8—H8	0.9300
C1—C6	1.378 (4)	C9—C10	1.358 (7)
C1—S1	1.750 (3)	C9—H9	0.9300
C2—C3	1.375 (5)	C10—C11	1.355 (8)
C2—H2	0.9300	C10—H10	0.9300
C3—C4	1.375 (6)	C11—C12	1.391 (6)
C3—H3	0.9300	C11—H11	0.9300
C4—C5	1.392 (6)	C12—H12	0.9300
C4—C13	1.492 (5)	C13—H13A	0.9600
C5—C6	1.349 (5)	C13—H13B	0.9600
C5—H5	0.9300	C13—H13C	0.9600
C6—H6	0.9300	N1—S1	1.633 (3)
C7—C12	1.373 (5)	N1—H1N	0.77 (4)
C7—C8	1.375 (5)	O1—S1	1.434 (2)
C7—N1	1.434 (4)	O2—S1	1.418 (2)
C8—C9	1.371 (5)		
			
C2—C1—C6	120.1 (3)	C8—C9—H9	119.1
C2—C1—S1	120.2 (2)	C11—C10—C9	119.2 (4)
C6—C1—S1	119.7 (3)	C11—C10—H10	120.4
C1—C2—C3	119.2 (3)	C9—C10—H10	120.4
C1—C2—H2	120.4	C10—C11—C12	121.0 (5)
C3—C2—H2	120.4	C10—C11—H11	119.5
C2—C3—C4	121.5 (4)	C12—C11—H11	119.5
C2—C3—H3	119.2	C7—C12—C11	118.6 (4)
C4—C3—H3	119.2	C7—C12—H12	120.7
C3—C4—C5	117.6 (3)	C11—C12—H12	120.7
C3—C4—C13	122.3 (5)	C4—C13—H13A	109.5
C5—C4—C13	120.1 (4)	C4—C13—H13B	109.5
C6—C5—C4	121.7 (3)	H13A—C13—H13B	109.5
C6—C5—H5	119.2	C4—C13—H13C	109.5
C4—C5—H5	119.2	H13A—C13—H13C	109.5
C5—C6—C1	119.9 (4)	H13B—C13—H13C	109.5
C5—C6—H6	120.1	C7—N1—S1	122.3 (2)
C1—C6—H6	120.1	C7—N1—H1N	109 (3)
C12—C7—C8	120.7 (3)	S1—N1—H1N	113 (3)
C12—C7—N1	117.2 (3)	O2—S1—O1	118.69 (15)
C8—C7—N1	122.0 (3)	O2—S1—N1	109.20 (17)
C9—C8—C7	118.7 (4)	O1—S1—N1	104.00 (16)
C9—C8—H8	120.7	O2—S1—C1	108.57 (15)
C7—C8—H8	120.7	O1—S1—C1	109.23 (16)
C10—C9—C8	121.8 (5)	N1—S1—C1	106.47 (14)
C10—C9—H9	119.1		
			
C6—C1—C2—C3	−0.9 (5)	C8—C7—C12—C11	−0.9 (6)
S1—C1—C2—C3	179.8 (3)	N1—C7—C12—C11	178.3 (4)
C1—C2—C3—C4	1.6 (6)	C10—C11—C12—C7	1.3 (7)
C2—C3—C4—C5	−1.0 (6)	C12—C7—N1—S1	135.5 (3)
C2—C3—C4—C13	−179.6 (4)	C8—C7—N1—S1	−45.3 (4)
C3—C4—C5—C6	−0.3 (5)	C7—N1—S1—O2	65.5 (3)
C13—C4—C5—C6	178.3 (4)	C7—N1—S1—O1	−166.9 (3)
C4—C5—C6—C1	1.0 (6)	C7—N1—S1—C1	−51.6 (3)
C2—C1—C6—C5	−0.3 (5)	C2—C1—S1—O2	−6.3 (3)
S1—C1—C6—C5	178.9 (3)	C6—C1—S1—O2	174.5 (2)
C12—C7—C8—C9	−0.1 (5)	C2—C1—S1—O1	−137.1 (3)
N1—C7—C8—C9	−179.2 (3)	C6—C1—S1—O1	43.7 (3)
C7—C8—C9—C10	0.7 (6)	C2—C1—S1—N1	111.2 (3)
C8—C9—C10—C11	−0.4 (7)	C6—C1—S1—N1	−68.0 (3)
C9—C10—C11—C12	−0.6 (7)		

### Hydrogen-bond geometry (Å, °)

**Table:**

*D*—H···*A*	*D*—H	H···*A*	*D*···*A*	*D*—H···*A*
N1—H1N···O1^i^	0.77 (4)	2.17 (5)	2.932 (4)	172 (4)

Symmetry codes: (i) −*x*+1, −*y*+1, −*z*+1.

## References

[bb1] Allen, F. H. (2002). *Acta Cryst.* B**58**, 380–388. 10.1107/s0108768102003890 12037359

[bb2] Enraf–Nonius (1996). *CAD-4-PC* Enraf–Nonius, Delft, The Netherlands.

[bb3] Gelbrich, T., Hursthouse, M. B. & Threlfall, T. L. (2007). *Acta Cryst.* B**63**, 621–632. 10.1107/S010876810701395X 17641433

[bb4] Gowda, B. T., Foro, S., Nirmala, P. G., Babitha, K. S. & Fuess, H. (2009*a*). *Acta Cryst.* E**65**, o476. 10.1107/S1600536809003845 PMC2968641 21582145

[bb5] Gowda, B. T., Foro, S., Nirmala, P. G., Babitha, K. S. & Fuess, H. (2009*b*). *Acta Cryst.* E**65**, o576. 10.1107/S160053680900573X PMC2968648 21582231

[bb6] Gowda, B. T., Foro, S., Nirmala, P. G., Terao, H. & Fuess, H. (2009). *Acta Cryst.* E**65**, o877. 10.1107/S1600536809010459 PMC2969044 21582588

[bb7] Gowda, B. T., Shetty, M. & Jayalakshmi, K. L. (2005). *Z. Naturforsch. Teil A*, **60**, 106–112.

[bb8] North, A. C. T., Phillips, D. C. & Mathews, F. S. (1968). *Acta Cryst.* A**24**, 351–359.

[bb9] Oh, I.-K., Kim, C.-J., Suh, I.-H. & Cho, S.-I. (1985). *Chung. Kwa. Yong. (Chung. J. Sci.)*, **12**, 67.

[bb10] Perlovich, G. L., Tkachev, V. V., Schaper, K.-J. & Raevsky, O. A. (2006). *Acta Cryst.* E**62**, o780–o782.

[bb11] Sheldrick, G. M. (2008). *Acta Cryst.* A**64**, 112–122. 10.1107/S0108767307043930 18156677

[bb12] Spek, A. L. (2009). *Acta Cryst.* D**65**, 148–155. 10.1107/S090744490804362X PMC2631630 19171970

[bb13] Stoe & Cie (1987). *REDU4* Stoe & Cie GmbH, Darmstadt, Germany.

